# Role of community health workers in type 2 diabetes mellitus self-management: A scoping review

**DOI:** 10.1371/journal.pone.0198424

**Published:** 2018-06-01

**Authors:** Bonaventure Amandi Egbujie, Peter Arthur Delobelle, Naomi Levitt, Thandi Puoane, David Sanders, Brian van Wyk

**Affiliations:** 1 School of Public Health, University of the Western Cape, Cape Town, South Africa; 2 Chronic Disease Initiative for Africa, Division of Diabetic Medicine and Endocrinology, Department of Medicine, University of Cape Town, Cape Town, South Africa; University of Botswana Faculty of Medicine, BOTSWANA

## Abstract

**Background:**

Globally the number of people with Type 2 diabetes mellitus (T2DM) has risen significantly over the last few decades. Aligned to this is a growing use of community health workers (CHWs) to deliver T2DM self-management support with good clinical outcomes especially in High Income Countries (HIC). Evidence and lessons from these interventions can be useful for Low- and Middle-Income countries (LMICs) such as South Africa that are experiencing a marked increase in T2DM prevalence.

**Objectives:**

This study aimed to examine how CHW have been utilized to support T2DM self-management globally, their preparation for and supervision to perform their functions.

**Method:**

The review was guided by a stepwise approach outlined in the framework for scoping reviews developed by Arksey and O’Malley. Peer reviewed scientific and grey literature was searched using a string of keywords, selecting English full-text articles published between 2000 and 2015. Articles were selected using inclusion criteria, charted and content analyzed.

**Results:**

1008 studies were identified of which 54 full text articles were selected. Most (53) of the selected studies were in HIC and targeted mostly minority populations in low resource settings. CHWs were mostly deployed to provide education, support, and advocacy. Structured curriculum based education was the most frequently reported service provided by CHWs to support T2DM self-management. Support services included informational, emotional, appraisal and instrumental support. Models of CHW care included facility linked nurse-led CHW coordination, facility-linked CHW led coordination and standalone CHW interventions without facility interaction.

**Conclusion:**

CHWs play several roles in T2DM self-management, including structured education, ongoing support and health system advocacy. Preparing and coordinating CHWs for these roles is crucial and needs further research and strengthening.

## Introduction

Type 2 diabetes mellitus (T2DM) has become one of the major causes of burden of disease globally. An estimated 422 million adults were living with the disease in 2014, compared with 108 million in 1980[1], representing a nearly quadruple increase within a period of thirty-four years. By 2035 the prevalence of T2DM is expected to increase by about 54% from the 2013 figures to about 592 million adults globally, increasing further to 642 million adults by 2040[2,3]. All regions of the world are affected, but low and middle income countries (LMIC), where over 70% of T2DM cases occur, suffer more[4]. About 12% of all health expenditure globally is estimated to be spent on T2DM and its associated complications alone with the majority of countries having to spend between 5–20% of their national health budget on the disease[3].

Sub-Sahara Africa (SSA) despite having the lowest T2DM prevalence presently is projected to have the highest rate of increase in T2DM cases of about 109% between 2015 and 2040[2,3].

With this epidemiological shift predicted for the region, contextually appropriate but low resource approaches are needed to manage the disease. Approaches that involve affected individuals and communities are key, because a large number of the population resides in rural areas with little access to healthcare facilities. The utilization of CHWs as a community-based strategy has been useful and effective in providing health promotion and prevention for patients with chronic diseases[5,6] Using CHWs to enhance patient and community management of T2DM is a potentially viable option and is reviewed in the current study. The review will focus on the roles they (CHW) play, including what they do, how they are prepared to perform these roles and what they influence (outcomes).

## Methods

This study was guided by the Arksey and O’Malley framework for conducting scoping reviews[7]. We initially defined and subsequently refined the research question based on the framework’s and other recommendations that broad scope research questions should be used to increase the amount of literature to be reviewed[7,8].

### Search strategy

We conducted a comprehensive literature search in the peer reviewed journal databases PubMed, CINAHL, COCHRANE, Scopus, SAGE, as well as grey literature repositories, Proquest Dissertation and Google scholar. The investigators chose three key constructs: ‘Type 2 diabetes mellitus,’ ‘community health workers’ and ‘self-management’ for the literature search based on a number of iterative meetings and discussions. The key constructs were searched using combinations which also included synonyms according to the database being searched to ensure that articles with related terms were equally identified. For PubMed search, the constructs were searched as MeSH terms, while for other databases, words such as ‘self-efficacy’ and ‘self-care’ were included as alternative to self-management, and ‘promotoras’, ‘promotores de salud’, and ‘community aides’ as alternative to CHWs.

The search was limited to articles published in English between January 2000 and December 2015 and identified abstracts were imported into Mendeley desktop software for review.

### Selecting relevant articles

**Fig 1:**
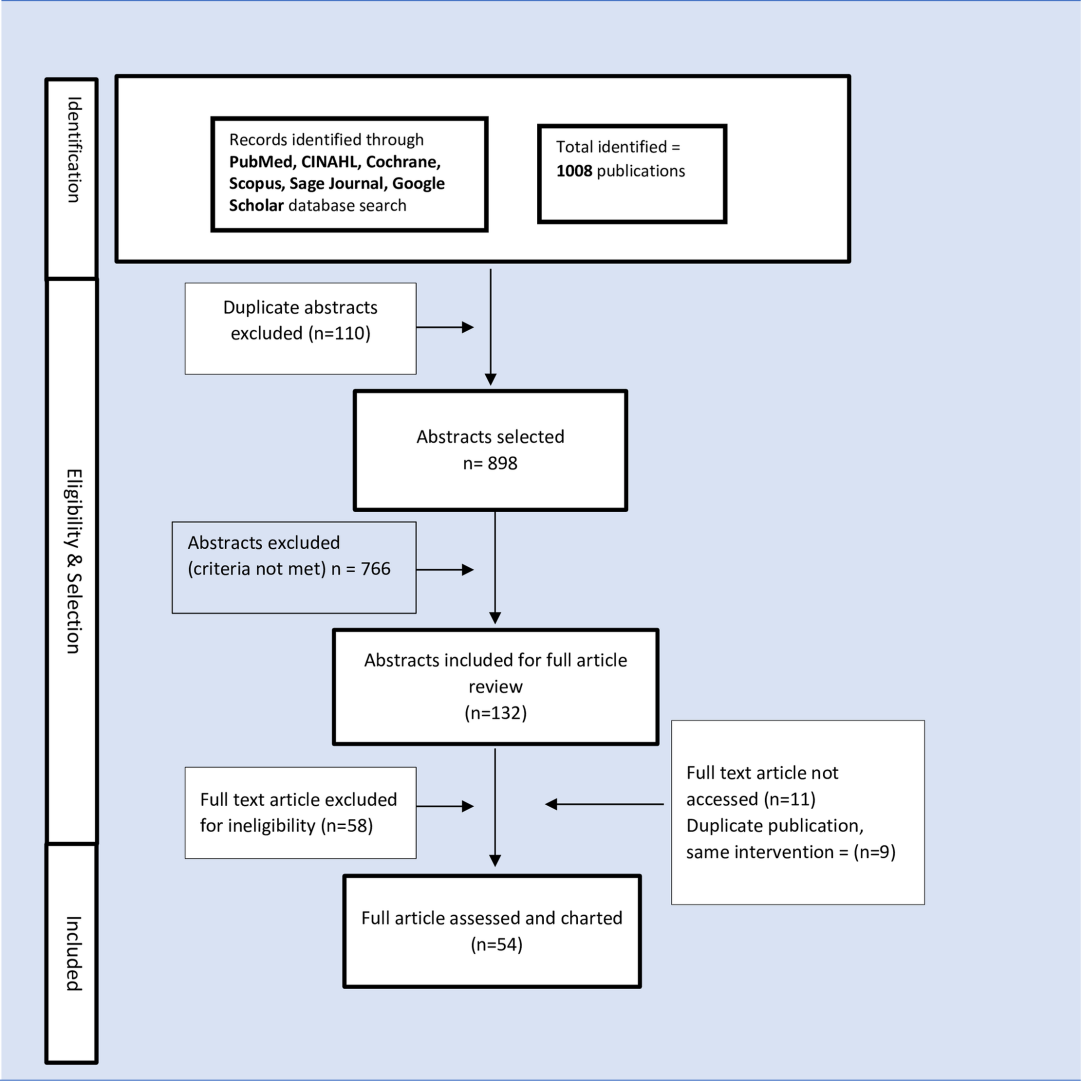
Diagram showing the flow process for identification and selection of eligible article in the scoping review.

Once retrieved from the literature search (as ‘hits’), we selected articles through a two-stage process. Firstly, all titles and abstracts were screened and eligible articles identified for full text retrieval. These retrieved full texts articles were subsequently screened for eligibility and selected according to set inclusion criteria. An article was included if the primary intervention focused on T2DM self-management or involved the use of CHWs or similar community-based non-professional health personnel. Exclusion criteria included literature reviews, peer rather than CHW interventions, commentaries, editorials, perspective articles or duplicates from already selected studies. Fig 1 shows the full process of article identification and selection for this studystudies. Fig 1 shows the full process of article identification and selection for this study.

### Charting selected articles

We charted articles to answer the research questions using a tool designed for that purpose. Charting was done by the first author with regular iteration among all co-authors, based on a review of the available information in each article, which was updated as new information emerged. We utilized both descriptive and narrative data analytical methods to glean information from articles[8].

### Collating, summarizing, and reporting the results

Combining the Arksey and O’Malley framework and the suggestions by Levac et al[8], we adapted the framework by breaking it into three stages to ensure that the collated reports answered a research question and created clear messages for the review. First, we conducted a descriptive, numerical analysis of the extent and nature of the current literature based on the search output presented in a narrative format, as charts and in table format. We then conducted thematic narrative synthesis in line with the research questions and finally discussed the implications of the first two outputs.

## Results

### Characteristics of selected articles

**Fig 2:**
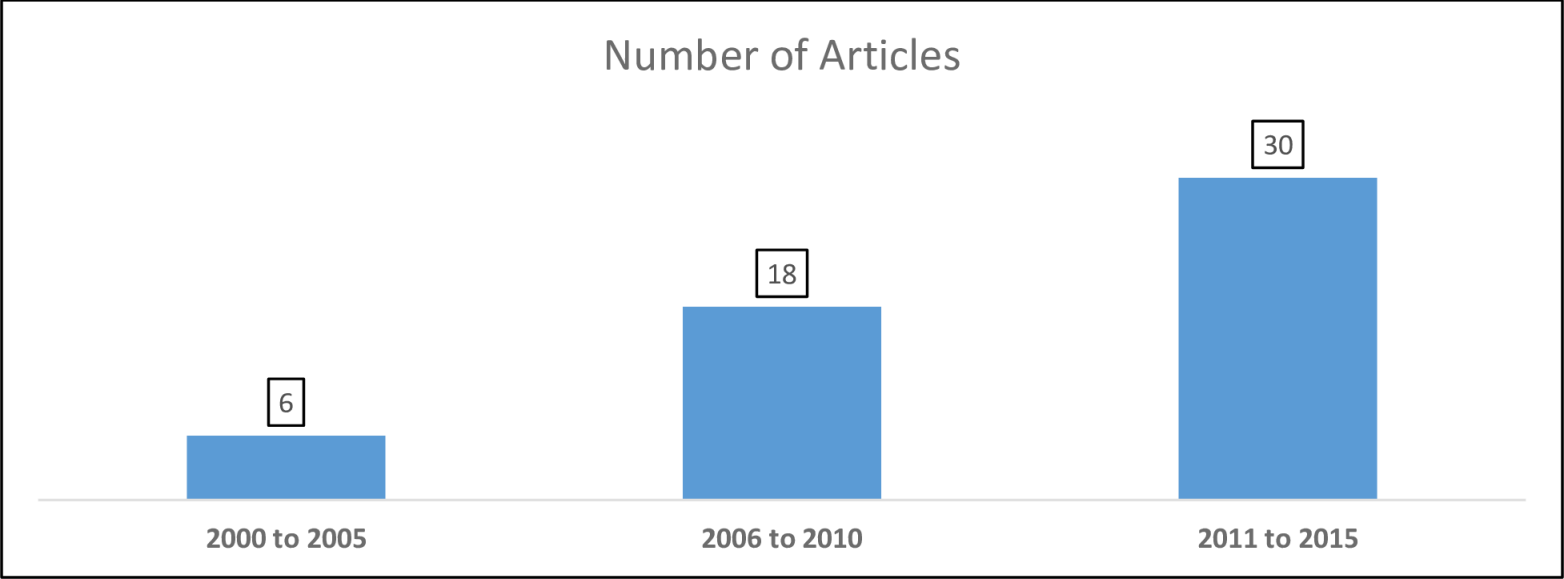
Diagram showing number of articles on CHW led T2DM self-management intervention in each five-year period between 2000 and 2015 among selected studies.

Full text of fifty-four (54) articles that met the inclusion criteria were reviewed. The summary of included articles can be seen in S1 Appendix. We found a five-fold increase in the number of relevant articles between the five year period 2011–2015 and the six year period 2000–2005, as shown in Fig 2.

**Fig 3:**
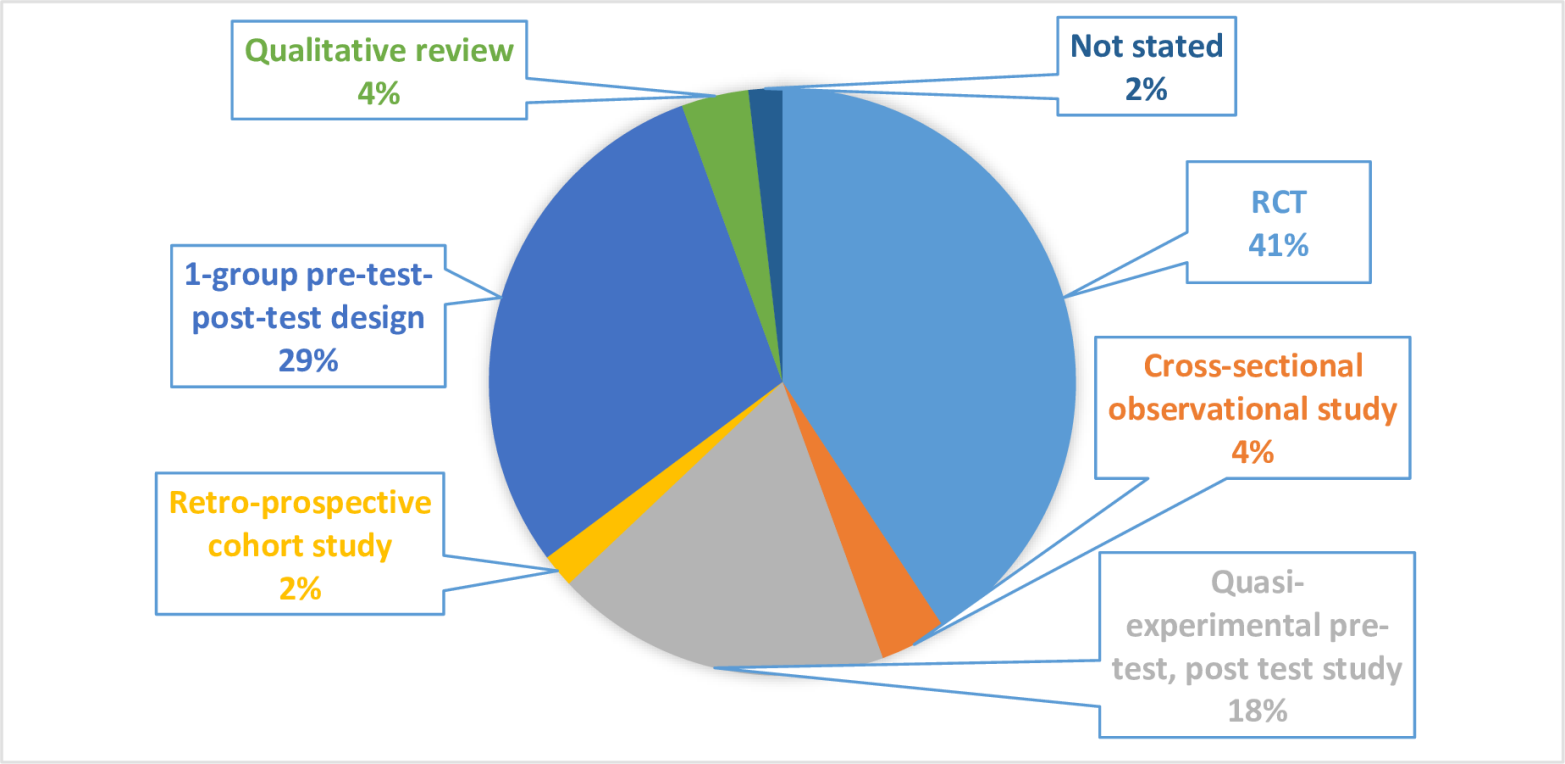
Diagram showing study designs commonly used to research CHW’s role in self-management of type 2 diabetes mellitus.

Randomized controlled trial (RCT) (n = 22), one group pre-test post-test (n = 16), and quasi-experimental (n = 10) designs were the most frequently reported methods to study CHW interventions for T2DM. Fig 3 shows the distribution of different study designs. Few studies used a cross sectional (n = 2) or qualitative (n = 2)_study design [9–13].

Fifty-two (52) articles described studies conducted in High Income Countries (HICs) and the US in particular (51). None of the studies were conducted in a low income country (LIC).

Minorities and disadvantaged groups were the most frequently targeted groups for CHW assisted T2DM self-management interventions. Hispanics, most of whom were Mexican Americans, made up more than 50% of this group, with African Americans also frequently studied. The study conducted in Australia that met our study criteria targeted Aboriginal people who also comprise a minority and disadvantaged group.

### Recruitment and selection

In all studies CHWs were recruited from the community where interventions took place (54 articles). These individuals are often known to be longstanding members of the intervention communities and being ‘bilingual’ was frequently used as a criteria for recruitment.

Academic qualification requirement for recruitment varied between studies. While high school diploma was frequently required for selection (16 articles), one article reported that there was no academic qualification requirement for selection as a CHW. Most studies (34 articles) failed to report the educational qualification of CHWs recruited for their intervention with 20 articles reporting that CHWs had at least a high school diploma.

### Training

We found that training duration varied significantly between studies ranging from a few hours to several months. Six studies (11%) reported that the training offered to CHWs lasted one week or less, and one article reported that the training duration was limited to four hours. Ten studies (19%) reported that training lasted more than a week with two reporting five and six months training durations. Thirteen studies (24%) indicated training duration lasting several hours (range 20–240 hours) but did not specify how these was spread in days, weeks or months. Curiously, most of the studies (24 or 44%) did not state training duration even when training was said to have been provided. No criteria were provided about how the duration of CHW training was decided upon in most of the articles. In the reported longest training duration (approximately six months), CHWs underwent training through a local health authority in order to be certified. From the reviewed studies, only 13 (24%) reported that CHWs were provided training updates in order to support their initial training. 35 studies (65%) did not state whether ongoing training was provided while 9 studies (16%) had no update training provided.

CHW trainings were frequently conducted by certified diabetes educators (CDE), intervention principal investigators (PI) or a research expert. The focus of PI led CHW training was often related to the study protocol. Few studies reported that CHWs were offered comprehensive multidisciplinary training by physicians, dieticians and CDE[14–16]. The use of an apprenticeship model for training, which refers to the coaching of newly recruited CHWs on the job by older CHWs, was reported in one study. We found limited information on the theoretical principle underlying CHW training. In one of the studies, adult learning technique was applied in training CHW.

[16–18]. From the review, training duration seemed to be associated with improved HbA1c and diabetes knowledge. The longer the training duration, the more likely study is to report improved HbA1c and diabetes knowledge as the study outcome. The only study that reported no improvement in diabetes knowledge had CHWs trained for a total duration of less than five hours.

## Roles played by CHWs

**Education.** Education was the most frequently reported (n = 44) service provided by CHWs in T2DM self-management. CHWs are commonly used as lay diabetes educators for T2DM patients[9,11,15,17,19–59]. The aim of education varied between studies but increasing patients’ knowledge was most frequently reported. CHWs also delivered stress management, meal preparation and planning, physical activity, problem solving, goal setting, as well as medication adherence education. None of the selected studies covered all the purposes mentioned above.

CHWs deliver education to T2DM patients in group, individually or both (9, 14 and 19 articles respectively). While individual education was usually delivered in patient’s homes, group education was delivered at various locations such as health facilities, churches, or other community settings. Education was often provided using paper tools but occasionally with electronic tools[17,33].

**Education curriculum**. CHWs deliver curriculum based education using manuals adapted from an existing document or documents developed purposely for the intervention. The American Diabetes Association (ADA) guidelines and American National Diabetes Education Program (NDEP) curriculum were the most frequently reported sources for CHW led education (n = 16). In other studies where curricula were developed for the study purpose, intervention designers used community-based participatory research (CBPR) with stakeholders including CHWs and other care providers.

### Support

In addition to their role as diabetes health educators, we found that CHWs were frequently deployed to provide support to T2DM patients (n = 42). This support was frequently provided in addition to health education but sometimes it served the sole purpose of their service. Support as used in this review varied between interventions, but generally belonged to one of the following four categories: emotional, appraisal, informational and instrumental/tangible support[60] These forms of support were usually delivered through one-on-one interaction at patients’ homes, follow-up phone conversations or group community settings in places such as churches.

CHWs most frequently provide informational support giving advice and information about community and other vital resources to patients (n = 30). Informational support refers only to non-curriculum based information on community resources for T2DM disease management. Four of these thirty studies reported that informational support was the only support intervention, while the remaining 26 studies reported that informational support was given in combination with other forms of support.

Twenty studies reported that appraisal support was given to patients at home in combination with other forms of support. Appraisal support referred to providing assistance or feedback to promote the conduct of self-management assessment (knowledge and behavior) by patients based on previously provided education.

CHWs also provided instrumental support to T2DM patients (n = 16). Instrumental support mostly involved helping patients to ‘navigate’ the health system and other systems that support the maintenance of healthy living such as social services[19,29], referrals and financial assistance. Other instrumental roles reported in the selected studies included helping with paperwork for services, accompanying patients, and arranging health services.

CHWs also provide critical emotional support to T2DM patients. Support services were often provided in combination and frequently tailored to each individual’s requirement.

### Advocacy

Advocacy is the third commonly reported role played by CHWs for T2DM self-management (10 articles)[15,22,43,61–67]. This role overlaps somewhat with the ‘instrumental support’ function as some articles reported referral support and setting up doctor’s appointments as advocacy. However, advocacy in this category refers to the role played by CHWs in helping participants communicate with their physician and health facility, ensuring that they receive good clinical services in line with stipulated guidelines. It includes helping patients to access health resources such as glucose strips, medication, and orthopedic shoes from health facilities, where accessing such commodities may take a longer period without help.

**Fig 4:**
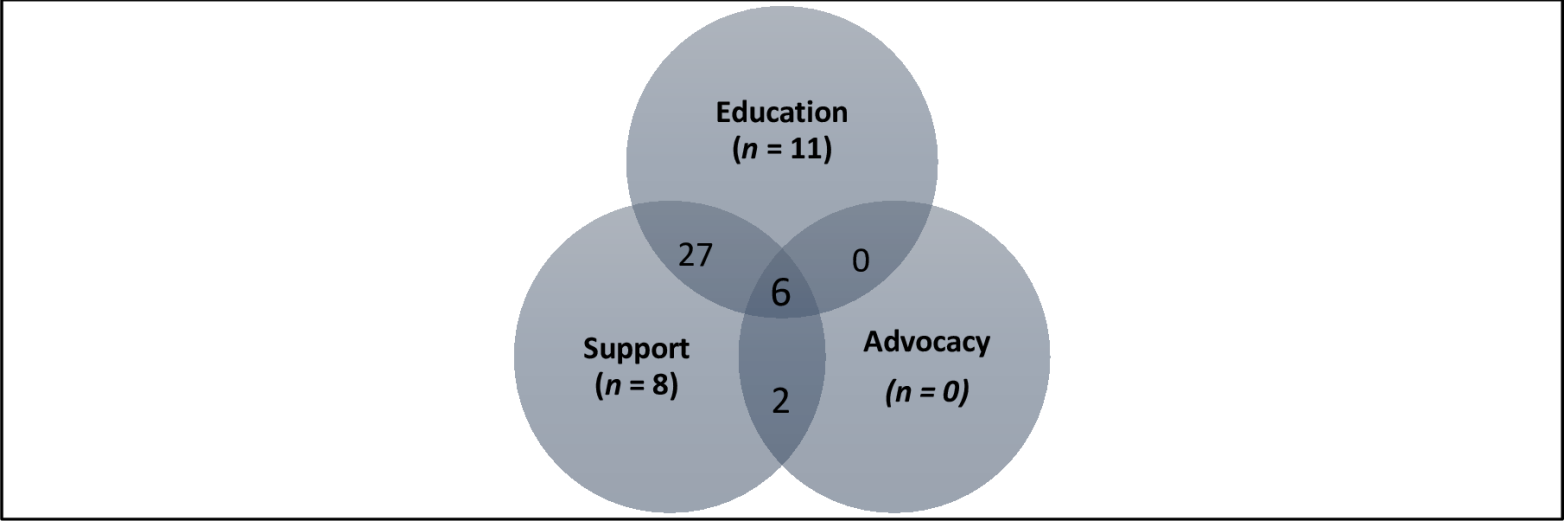
Venn diagram showing distribution of CHWs role in T2DM in selected articles, *n =* number of articles.

The selected articles described CHWs roles as described above in different combinations, as shown in Fig 4. We found that a combination of Education and support activities is the most frequently reported approach and is more likely to lead to improvement in HbA1c as well as diabetes knowledge compared to other combinatiosn of roles. This combination is also found to lead to better outcomes compared to any of the roles alone. When a single role is used, we found that Education only approach leads to the best outcomes. The review also showed that education only intervention using CHW is less likely to result in weight or BMI reduction compared to combination of education and support activities by CHW.

### Coordination

As the selected studies indicate, three broad models of CHW coordination are frequently used in T2DM self-management interventions. In the first model, CHWs are coordinated by intervention managers or research investigators, who are usually not employed by the health facility where the intervention is situated and where coordination is essential for intervention or research implementation. There is no direct interaction between CHWs and usual patient care providers in this model (n = 17). In the second model, CHWs are supervised and coordinated by a nurse or physician in the facility where the patients receive their clinical services (n = 27). CHWs communicate routinely with these usual care providers. Studies where this model was used, reported that CHWs were mostly employed by the health facility and fully integrated and hence coordinated and supervised in the existing health system. Among the twenty-seven interventions where nurses coordinated CHWs, they had designated T2DM intervention activities (n = 7) or performed a regular duty in the facility in addition to facilitating CHW T2DM activities (n = 20). Where a nurse was dedicated solely to T2DM intervention by coordinating CHWs, such nurse was often referred to as nurse-case manager (NCM).

One study looked at the effectiveness of the different models of coordination mentioned above and showed that the NCM model of coordination was more effective than interventions where CHWs were not coordinated by nurses or where nurses alone provided T2DM services[68]. In the third model of coordination, a more experienced CHW is used to coordinate other CHWs. Often designated as the senior CHW, this person communicates with health facilities to facilitate the work of CHWs.

### Targeted outcomes

**Fig 5:**
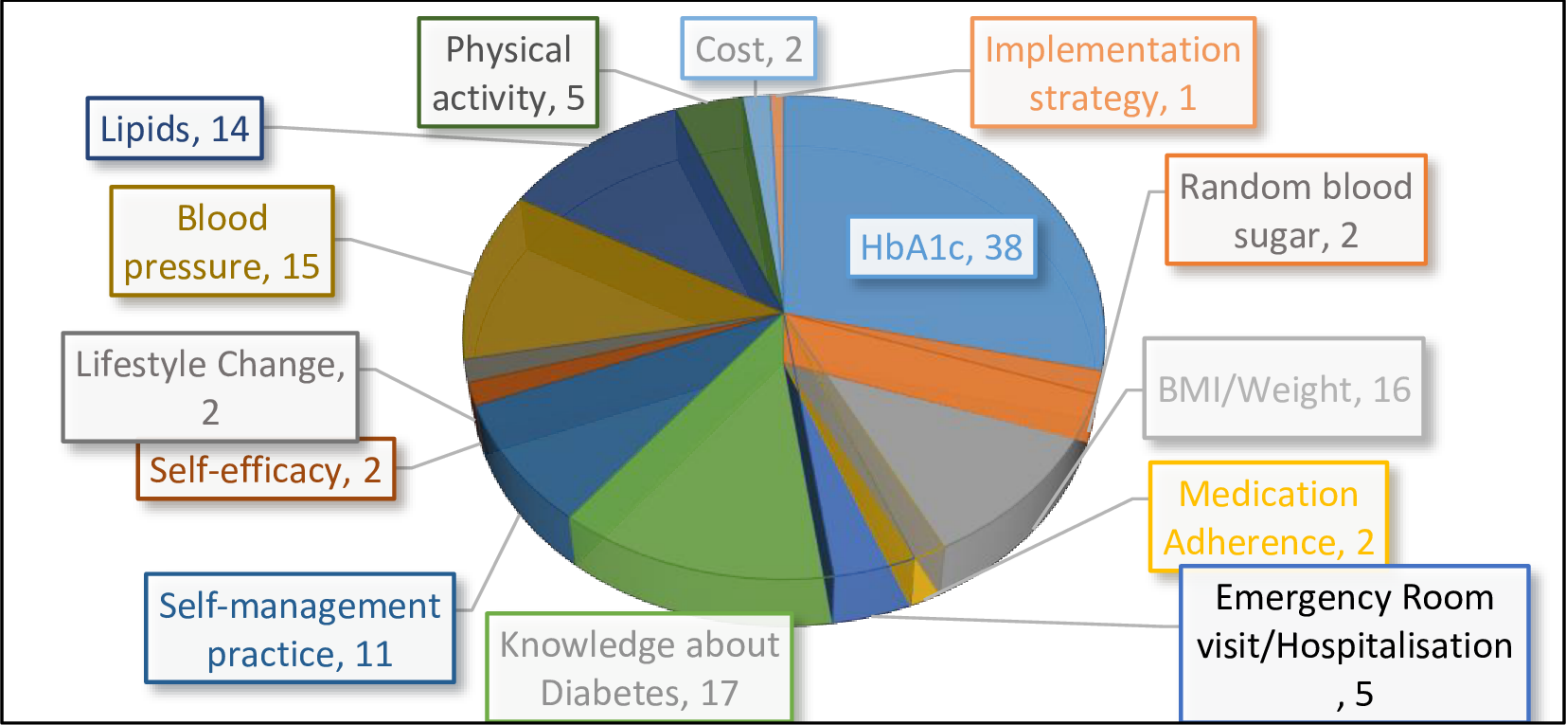
Diagram showing outcomes frequently targeted and reported in CHW supported self-management of type 2 Diabetes mellitus study.

Blood sugar control and blood lipid levels were the two most frequently measured outcomes in CHW led T2DM self-management interventions in our review (Fig 5). Thirty-eight (70%) of the studies reported that glycosylated hemoglobin (HbA1c) was the biomarker used to monitor blood sugar control and measure the success of intervention. One study reported using random blood sugar to measure blood sugar control among T2DM patients[10]. In studies that used HbA1c as primary outcome measure, a few reported improvement in values for patients whose baseline HbA1C were much higher than the normal (closer to 10%), with very little or no gains for patients whose values were close to the normal range (<7%)[56,69].

Fourteen studies (24%) measured the lipid profile of participants as a primary or secondary outcome. Fifteen articles reported blood pressure checks in addition to blood sugar level as a measure of effectiveness of intervention, while 16 articles reported that BMI was measured to monitor CHW effectiveness in T2DM self-management. Other studies reported using physical activity, medication adherence, number of clinic visits, fruit and/or vegetable intake as the measure of success.

Apart from the biophysical outcomes mentioned above, seventeen articles (31%) reported that T2DM knowledge was the key outcome measure. Other studies reported improved self-management behavior or self-efficacy as outcome measure using standardized tools. The self-management tools commonly reported in our review included the ‘Summary of Diabetes Self-care Activities’ tool[55,57,64,70,71], the Morisky Medication Adherence Scale[19,71] and Lorig Self-efficacy Scale[57,72]. It is important to mention that although all studies targeted self-management as an outcome, only seven studies reported the use of standardized scales to measure self-efficacy or self-management.

## Discussion

We conducted this study to map the available literature and evidence related to CHWs’ roles in T2DM self-management interventions globally.

The roles increasingly played by CHWs in T2DM self-management are most prominent in HIC, in particular the US. Based on the findings from our review we have categorized these roles into the triad of education, support and advocacy (ESA). Palmas et al (2015) in a systematic review of CHW interventions similarly identified education, support and advocacy as the major roles played by CHWs[73]. In the US, the National Community Health Advisor Study through a national survey listed seven basic roles for CHWs including cultural mediation, informal counselling & social support, education, advocacy, ensuring people get services they need, capacity building and direct service provision[74]. Findley et al (2012) in a New York State survey among CHWs and employers identified five basic roles played by CHWs including advocacy, education, community outreach, referral and cultural bridging[75]. Norris et al (2006) found a great variability in the roles played by CHWs in diabetes care[76].

As part of the recommendation for the “1 million CHWs in sub-Saharan Africa by 2015”, Singh and Sachs (2013) noted that CHWs in the ‘Millennium Village project’ essentially played three roles viz. education, pre-approved clinical services and referral[77]. Our categorization of CHW roles represents a functional approach to relaying their use in self-management interventions and is not different from the classifications described above. Our classification is however based on CHWs’ role in diabetes self-management only, while some of the roles mentioned in studies above are for several other chronic conditions.

In our review CHWs were most commonly used as diabetes educators for T2DM intervention. Diabetes education is not always available in every country and even when available, patients do not always have access and therefore are not reached with this critical component of care[43,67]. As educators, CHWs are used to bridge this care gap by providing accessible education to people in their preferred locations, in particular focusing on disadvantaged populations.

In addition to education, CHWs are also used to provide social support which can be emotional, informational, appraisal and tangible in nature[39,62]. The definition of this support varied across studies, although it is commonly recognized as being crucial to successful self-management. As noted by Davis et al (2007), improvements in diabetic control following CHW self-management education intervention begins to decline six months after the end of intervention, [15,43,76] and lifetime follow-up and support may be required[78], especially in settings where there is a lack of professional health care providers.

CHWs also provide advocacy services to T2DM patients. This is the least reported role because advocacy had different meanings in different studies. Culica et al (2007) and Ingram et al (2007) considered activities such as filling papers and referring T2DM patients to clinics as advocacy, but we have classified the latter as part of instrumental or tangible support. Advocacy has not been comprehensively explored by diabetes program designers and CHWs have been reported to be on the periphery of formal health systems[79], without much integration, which may have hampered their role as advocates.

CHWs often undertake different roles in T2DM self-management, with studies showing this to be an increasing trend between 2000 and 2015. Education and support alone is not sufficient to provide self-management capabilities to T2DM patients. Cummings et al (2013) in a randomized controlled trial of education alone compared with education alongside other roles for CHWs found and suggested that programs should not only focus on didactic diabetes education, but should include other support activities that increase coping skills[29].

Coordination and supervision of CHWs in their roles is crucial for success or failure[79]. We found different coordination models which either made CHWs outsiders or insiders to patient routine care. Models that integrate CHW coordination within the health system and give CHWs access to regular patient care were found to be effective. Lehmann and Sanders (2007) noted that CHWs often work peripherally and are not directly coordinated by the health system, which can make it difficult to fully participate in patient management. There is evidence of improved clinical outcomes for T2DM self-management interventions when a CHW works with, and is coordinated, by a health facility linked nurse for program delivery[31,61,68].

Preparing CHWs to play their role in T2DM self-management is a critical yet often neglected component of intervention design. Insufficient training undermines successful implementation of well-intended CHW interventions[10]. Just as CHW training duration and content are critical, the type and quality of training providers is also critical to successful knowledge transfer. CDEs were often tasked with training CHWs in the reported studies, although few reported that CHWs were trained by endocrinologists, CDE, mental health professionals, and nutritionists in a complete training package. Training in study policies and procedures, technical skills, and diabetes education alone may not provide CHWs enough capacity to perform the role of health educators, and more may be required[80]. The involvement of a multidisciplinary team of medical and health promotion professionals in the training of CHWs may represent the optimal way to ensure quality and consistency. Of equal significance is the provision of ongoing training to CHWs. Most studies in this review lasted between 6–12 months and did not identify the need for ongoing training. For interventions at population level that will potentially last several years, ongoing training will however be necessary.

In the reviewed literature, HbA1c was the most frequently used measure of success in CHW led T2DM self-management interventions. HbA1c is the recommended standard test for glycemic monitoring in diabetes as it measures average plasma glucose over 2–3 months[81,82] and is thus the appropriate test if glycaemic control is the preferred outcome measure. This suggests that CHWs are used to target and focus on chronic glycemic control rather than acute control in T2DM management. It is important to mention that in HIC, HbA1c tests may be freely available as a routine diabetes investigation, but for many LMIC, routine access to HbA1c is limited. Thus, in a study setting, using HbA1c as an outcome measure in LMIC is quite possible, but it would be more difficult in routine care in rural or primary care settings where cost and access to even point of care HbA1c machines may be limited. Bennet et al recognized such challenges to the use of Hb1Ac in poorer countries[82]. A good knowledge of the interaction between hemoglobin levels and HbA1c is also important since different hemoglobin levels could affect the interpretation of any HbA1c result obtained[83,84]. Having said these, it is important to recognize that not all patients will experience huge benefit in terms of reduction in HbA1c value when supported by CHW no matter the roles or combination of roles. There is therefore the need for triaging and selection criteria for the clients who will most likely benefit from CHW support as far as HbA1c reduction is concerned.

T2DM disease knowledge was also a frequently reported primary or secondary outcome in our review. To improve their self-efficacy, patients will require good knowledge of T2DM disease as well as other self-management skills, including goal setting and coping skills. The ultimate goal of T2DM education is to improve self-efficacy in individuals, and as such, any education falling short of this cannot be said to be adequate. The measures in our review were mostly related to T2DM knowledge and not inclusive of self-management or self-efficacy. Non-inclusion of self-efficacy as part of a CHW led diabetes education intervention outcome could make it difficult to evaluate the actual impact of such intervention or the pathways through which any noted changes occurred. As observed in our review, increased T2DM knowledge do not always translate into improved glycemic control[48,56,64], which could be due to the fact that increased knowledge did not result in improved self-management. Studies have observed that increased knowledge does not necessarily translate into changes in self-management behaviour[29,37], and there is a need for further research to answer questions related to the association or lack of it between improved T2DM knowledge, self-efficacy and ultimately glycemic control. Outcome measures in diabetes education intervention should hence not only speak to increased diabetes knowledge but also include self-management and self-efficacy measures.

## Key lessons

Patients with poorly controlled blood sugar are more likely to benefit from CHW support compared to patients with better glycaemic control. This suggests that there could be usefulness to triaging of patients most at risk for the sake of providing CHW support. Secondly, CHW training duration may have influence on the quality of support they provide and ultimately affect the clinical target outcome. Thirdly, Improvement in blood sugar control does not always align with increased diabetes knowledge or self-management practice among T2DM patient as shown in our reviewed studies, which suggests existence of other mediators or pathways through which self-management support leads to improved clinical outcome.

## Recommendations for research

Training methodology that most appriopriately capacitates CHW for T2DM self-management support was not clear from the review. More studies are required to establish how best to train CHW for their role in T2DM support. This includes who should provide the training and for how long. More studies will be required to answer question on whether triaging T2DM patients for CHW support will be beneficial overall or less not. Furthermore research into the relationship between diabetes self-management support activities (education, support, advocacy), diabetes knowledge, self- efficacy and improved outcomes will provide more information on what works and should be pursued further.

## Limitations

Although we made stringent efforts to ensure that all available literature was identified and included, we recognize that some articles may have been missed. We searched for articles in three peer reviewed journal databases and an online data source and used two databases to search for the full text of selected abstracts. It is possible that some articles have been missed, especially in the grey literature. We are also cautious about interpreting the findings from this study since most selected studies came from one country, the US. It could however be argued that the use of CHWs for T2DM self-management is most common in the US and hence most research publications will be from this country. Further to this, abstract and full text selection was done by one author rather than by two independent researchers as recommended by Arksey and O’Malley. This could result in researcher bias in including or excluding articles from full review. Iteration between all the authors however ensured rigorous article selection and charting. Despite the above reported limitations, we are confident that the findings from this study are a true reflection of the roles CHWs play in T2DM self-management intervention.

## Conclusion

CHWs play several useful roles in T2DM self-management that includes structured education, ongoing support and health system advocacy. Most of the available evidence however come from HICs with very little from LMICs. Preparing and coordinating CHWs for these roles is essential for success and will need further research and strengthening. Training models that deliver comprehensive knowledge and enhance CHWs’ capacity to deliver T2DM self-management support is lacking and needs to be developed. Further studies should attempt to explore the mediating link between self-efficacy and self-management of T2DM when CHWs are used.

## Supporting information

S1 Appendix Summary of the characteristics of selected studies I. (PDF)

S2 Appendix Summary of the characteristics of selected studies II. (PDF)
