# Supplementary material for: Role of community health workers in type 2 diabetes mellitus self-management: A scoping review
Source: PLoS One. 2018 Jun 1;13(6):e0198424. doi: 10.1371/journal.pone.0198424 (PMC5983553; doi:10.1371/journal.pone.0198424)
Supplement: S1 Appendix — (PDF) [file pone.0198424.s001.pdf]

S1 Appendix

Short Title	Title	Aim	Study design	Target Population	Theoretical Framework	Study duration	Training duration	Follow up training
Brown (2002)	Culturally competent diabetes self-management education for Mexican Americans: the Starr County border health initiative.	To determine the effects of a culturally competent diabetes self-management intervention in Mexican Americans with type 2 diabetes.	• RCT	• Hispanic Americans *Mexican Americans*		• 12 months	• Not stated	• not stated
Gary (2003)	Randomized controlled trial of the effects of nurse case manager and community health worker interventions on risk factors for diabetes-related complications in urban African Americans	To determine the effects of nurse case manager and community health worker interventions on risk factors for diabetes-related complications in urban African Americans	• RCT	• African Americans *urban African Americans*	• Eco-behavioural/ Ecological Action Model<br>• Learning Theory	• 24 months		• not stated
Higgins (2003)	The Promotora Telemedicine Project : Combining Technology and Cultural Sensitivity to Improve Diabetes Care in a Medically Underserved Community	to test the hypothesis that new technology can be combined with culturally sensitive programs to improve access to high-quality care and to reduce the cost of diabetes care	• Quasi-experimental pre-test, post test study	• Hispanics and African Americans	• Not stated	• Not stated	• Not stated	• None provided
Gary (2004)	A randomized controlled trial of the effects of nurse case manager and community health worker team interventions in urban African-Americans with type 2 diabetes	to determine the effectiveness and cost-effectiveness of primary care and community-oriented interventions in managing HbA1c, blood pressure, and lipids, and reducing hospitalizations and emergency room visits over 2 years	• RCT	• African Americans *urban African Americans*	*Precede–Proceed model*	• 24 months	• >1 month<br>*6 weeks*	
Sul (2004)	The Involvement of Lay Educators in the Diabetic Control of Type 2 Diabetic Patients	to assess the effectiveness of introducing lay health educators into the community to improve diabetic control in Type 2 diabetic patients	• RCT	• Asians *Malaysian*	• Not stated	• 6 months	• <5 hours	• not stated
Two (2005)	Racial and Ethnic Approaches to Community Health (REACH) Detroit partnership: improving diabetes-related outcomes among African American and Latino adults.	to determine the effects of a community-based, culturally tailored diabetes lifestyle intervention on risk factors for diabetes complications among African Americans and Latinos with type 2 diabetes	• Quasi-experimental pre-test, post test study	• Hispanics and African Americans	• Cognitive Theory	• Not stated	• >1 month<br><br>*FHA trained for 10 weeks on FHA training programme, also on patient empowerment approaches.*	

Ingram (2007)	The impact of promotoras on social support and glycemic control among members of a farmworker community on the US-Mexico border.	to describe the effect of a promotora-driven intervention to build social support as a means to affect self-management behaviors and clinical outcomes in a farmworker community on the US-Mexico border	• 1-group pre-test-post-test design	• Hispanic Americans<br>*Mexican Americans*	• Unclear	• 12 months	• Not stated	• not stated
Lujan (2007)	Promotora diabetes intervention for Mexican Americans.	to determine the effectiveness of an intervention led by promotoras (community lay workers) on the glycemic control, diabetes knowledge, and diabetes health beliefs of Mexican Americans with type 2 diabetes	• RCT	• Hispanic Americans<br>*Mexican Americans*	• Community-oriented model<br>*Community empowerment*	• 6 months	*60 hour training on diabetes self-management*	• None provided
Hill-Briggs (2007)	Training community health workers as diabetes educators for urban African Americans: value added using participatory methods	To describe procedures used to recruit, train, and evaluate CHWs in Project Sugar 2, a randomized controlled trial of a nurse case manager and CHW team intervention designed to improve diabetes care and control in urban African Americans with type 2 diabetes	• Not stated	• African Americans		• Not stated	*6 phases, >240 hours of training*	• not stated
Thompson (2007)	Advancing diabetes self-management in the Mexican American population: a community health worker model in a primary care setting.	to pilot test the effectiveness of health promoters trained in the transtheoretical model of change to provide diabetes management education and support to Mexican Americans in a primary care setting	• 1-group pre-test-post-test design	• Hispanic Americans<br>*Mexica americans*	*transtheoretical model of change*	• 12 months	• >1 month<br>*10 sessions of general training and 30 hours of diabetes management and transtheoritical model*	*biweekly meetings provided ongoing instruction and support*
Davis (2007)	Teaching How, Not What: The Contributions of Community Health Workers to Diabetes Self-Management	to describe ways in which com- munity health workers (CHWs) are used in various clinic and community settings to support diabetes self-management	• Qualitative review	• Hispanic Americans	• Not stated	• Not stated<br>• Not applicable	• Not stated	• not stated
Liebman (2007)	Establishing diabetes self-management in a community health center serving low-income Latinos.	to describe the implementation of the chronic care model for Latinos with diabetes	• 1-group pre-test-post-test design	• Hispanic Americans	• Not stated	• More than 24 months	• Not stated	• not stated
Vincent (2007)	A feasibility study of a culturally tailored diabetes intervention for Mexican Americans.	to test the feasibility and examine the effects of a culturally tailored intervention for African American men with type 2 diabetes on outcomes of self-management	• 1-group pre-test-post-test design			• Less than 6 months<br>*3 months*		

Culica (2007)	CoDE: Community Diabetes Education for uninsured Mexican Americans.	To describe the structure, patient acceptance, and costs of a one-to-one educational model delivered by a community health worker in a community clinic serving the uninsured	• Quasi-experimental pre-test, post test study	• Hispanic Americans<br>*Mexican Americans*	• Not stated	• 12 months	*27 hours of training provided by Certified Diabetic educator, dietitian and Endocrinologist*	
Beckham (2008)	Diabetes management: utilizing community health workers in a Hawaiian/Samoan population	To examine the effectiveness of CHWs on diabetes management among a population with primarily Native Hawaiian and Samoan ethnic minority participants with HbA1c greater than 10%	• Quasi-experimental pre-test, post test study	• American Samoa	• Not stated	• 12 months	• >1 month<br>*6 months*	*Weekly and then monthly training for the duration of the project.*
Sixta (2008)	Texas-Mexico Border Intervention by Promotores for Patients With Type 2 Diabetes	to evaluate the impact of a promotores-led diabetes self-management program by comparing the outcomes (knowledge, beliefs, and HbA1c level) of Mexican American patients with type 2 diabetes	• RCT	• Hispanic Americans<br>*Mexican Americans*		• 6 months	• Not stated	• not stated
Liebman (2008)	Quality Improvement in Diabetes Care Using Community Health Workers	to improve diabetes care among limited English-proficient (LEP) Latino patients	• Quasi-experimental pre-test, post test study	• Hispanic Americans<br>*Mostly hispanics*	• Unclear	• Unclear	• 8 days - 1 month	• not stated
McElmurry (2009)	Implementation, outcomes, and lessons learned from a collaborative primary health care program to improve diabetes care among urban Latino populations.	To evaluate the relative effectiveness of a CHW intervention among Hispanic persons with newly diagnosed type 2 diabetes, as compared with usual clinic practice	• Quasi-experimental pre-test, post test study	• Hispanic Americans<br>*Urban latinos*	• Not stated	• Not stated	• Not stated	*several-month period of on-the-job-training and additional formal learning experiences*
Babamoto (2009)	Improving Diabetes Care and Health Measures Among Hispanics Using Community Health Workers: Results From a Randomized Controlled Trial	To pilot test the efficacy of a culturally tailored diabetes self-management social support intervention for Mexican American adults with Type 2 diabetes (T2DM)	• RCT	• Hispanic Americans	*transtheoretical (stages of change) model*	• 6 months	*6 weeks training on Diabetes standards, self management startegies, health beliefs*	• not stated
McEwen	Type 2 diabetes self-management social support intervention at the U.S.-Mexico border.	To improve self-management among patients with type 2 diabetes incorporating CHWs as members of a clinical team	• 1-group pre-test-post-test design	• Hispanic Americans<br>*Mexican Americans*	• Not stated	• 6 months	• >1 month<br>*8 weeks (24 hours)*	*Ongoing 1-hr monthly training*

Otero-sabogal (2010)	Physician – community health worker partnering to support diabetes self- management in primary care	to explore the role of the promotora de salud (health promoter) who provided diabetes self-management education to Puerto Rican diabetics in her community	• Quasi-experimental pre-test, post test study	• Hispanic Americans *Low income latino*	• Not stated	• 12 months	• Not stated	• not stated
Deitrick (2010)	Understanding the Role of the Promotora in a Latino Diabetes Education Program	To conduct a diabetes education program delivered by community health workers in community settings; and evaluate its effectiveness in improving glycemic control and self-management skills in Hispanic/Latinos with type 2 diabetes	• Qualitative review *focus group discussion*	• Hispanic Americans *Puerto ricans*	• Not stated	• Not stated		
Castillo (2010)	Community-based Diabetes Education for Latinos The Diabetes Empowerment Education Program	To test the feasibility of implementing a group health education and intervention model to reduce the incidence of diabetes and obesity among African American men; (2) improve regular access to and utilization of health care services and community supportive resources to promote healthy lifestyles among African American men; and (3) build community networks and capacity for advocacy and addressing some of the health needs of African American men	• 1-group pre-test-post-test design	• Hispanic Americans	*principles of adult education and empowerment theory*	• Less than 6 months	*20 hours of TOT*	
Treadwell (2010)	Addressing obesity and diabetes among African American men: examination of a community-based model of prevention.	To evaluate the effectiveness of a comprehensive program of CVD risk reduction delivered by nurse practitioner (NP)/community health worker (CHW) teams versus enhanced usual care	• 1-group pre-test-post-test design	• African Americans	• Not stated	• Less than 6 months	• Not stated	• not stated
Allen (2011)	COACH trial: a randomized controlled trial of nurse practitioner/community health worker cardiovascular disease risk reduction in urban community health centers: rationale and design.	To test the effectiveness of a culturally tailored, behavioral theory-based community health worker intervention for improving glycemic control	• RCT	• Hispanics and African Americans *White and non-whites*	• Motivational Interviewing	• 12 months	• Not stated	
Spencer (2011)	Effectiveness of a Community Health Worker Intervention Among African American and Latino Adults With Type 2 Diabetes: A Randomized Controlled Trial	to determine the impact of Community Health Workers (promotoras de salud) on the self-management practices of people with diabetes on the U.S.-Mexico Border	• RCT	• Hispanics and African Americans	*Empowerment Theory, socioecologica model*	• 6 months	*More than 80 hours*	• not stated

Ryabov (2011)	The impact of community health workers on behavioral outcomes and glycemic control of diabetes patients on the U.S.-Mexico border	To estimate the long-term cost-effectiveness of a lifestyle modification program led by community health workers (CHWs) for low-income Hispanic adults with type 2 diabetes.	• RCT	• Hispanic Americans *Mexican Americans*	• Not stated	• 24 months	• Not stated	• not stated
Brown (2012)	Cost-effectiveness analysis of a community health worker intervention for low-income Hispanic adults with diabetes.	Examine the effectiveness of using community health workers (CHWs) to support nurse-led diabetes self-management education (DSME) with medically underserved clients	• Quasi-experimental pre-test, post test study	• Hispanic Americans	• Not stated	• 18 months	• Not stated	• not stated
Kegley (2012)	Role of Community Health Workers in Improving Diabetes Outcomes	CHW-led diabetes self-management education program targeting Hispanic patients	• 1-group pre-test-post-test design	• Hispanics and African Americans	• Not stated	• Unclear	• Not stated *CHW trained but duration not stated*	• not stated
Walton (2012)	Reducing Diabetes Disparities Through the Implementation of a Community Health Worker–Led Diabetes Self-Management Education Program	to deliver a culturally relevant diabetes education program to a Hispanic population at a migrant clinic	• 1-group pre-test-post-test design	• Hispanic Americans	• Learning Theory *Adult learning Theory of knowles*	• 12 months	*160 hours of training asTexas certified CHW and another 50 hours training on Diabetes education and project protocol.*	*35 hours of continuing education delivered by certified diabetes educators, physicians, a dietician, and a CHW certified trainer.*
Valen (2012)	An innovative approach to diabetes education for a hispanic population utilizing community health workers	to answer key implementation questions from our translation research with a primary care-based, nurse-community health worker (CHW) team intervention to support type 2 diabetes self-management	• 1-group pre-test-post-test design	• Hispanic Americans		• Unclear	• 2 - 7 days *12 hours of initial training*	*16 hours of ongoing training in between the program*
DePue	Implementation of a culturally tailored diabetes intervention with community health workers in American Samoa.	to determine the impact of a culturally tailored diabetes education program led by a community health worker (CHW) on the HbA1c, blood pressure, body mass index (BMI) and lipid status of uninsured Mexican Americans with diabetes	• Cross-sectional observational study	• American Samoa	• Unclear	• 12 months	• 2 - 7 days *extensive training on diabetes*	• not stated *new recruits training is by apprenticeship model*

Prezio (2013)	Community Diabetes Education (CoDE) for uninsured Mexican Americans: a randomized controlled trial of a culturally tailored diabetes education and management	to evaluate the effectiveness of a Community Health Worker (CHW)-led diabetes self-management education (DSME) program and to understand how CHWs and primary care providers (PCPs) work together to provide comprehensive diabetes care	• RCT	• Hispanic Americans *uninsured Mexican Americans*	• Cognitive Theory	• 12 months		
Collinsworth (2013)	Effectiveness of a community health worker-led diabetes self-management education program and implications for CHW involvement in care coordination strategies.	To evaluate the effectiveness of a culturally adapted, primary care–based nurse–community health worker (CHW) team intervention to support diabetes self-management on diabetes control and other biologic measures	• 1-group pre-test-post-test design *mixed method was applied*	• Hispanic Americans	• Not stated	• 12 months	• Not stated	• not stated
De Pue (2013)	Nurse–Community HealthWorker Team Improves Diabetes Care in American Samoa	To describe the application of CBPR and UCD principles to the development of iDecide/Decido, an interactive, tailored, web-based diabetes medication education and decision support tool delivered by community health workers (CHWs) to African American and Latino participants with diabetes	• RCT	• American Samoa	• Not stated	• 12 months	*Extensive training but duration not mentioned. Apprenticeship model of training used for new recruits*	• not stated
Henderson (2013)	Community-based participatory research and user-centered design in a diabetes medication information and decision tool.	examines the outcomes of a pilot CHW programme to improve the management of hypertension and diabetes	• Quasi-experimental pre-test, post test study	• Hispanics and African Americans	• Not stated	• Not stated		
Ndou (2013)	A rapid assessment of a community health worker pilot programme to improve the management of hypertension and diabetes in Emfuleni sub-district of Gauteng Province, South Africa.	evaluated the effectiveness of the Salud y Bienestar program to deliver diabetes education in the Hispanic population in the United States.	• Cross-sectional observational study	• Africans *South Africans*		• Not stated	• >1 month *14 weeks of training on home based care provision, health promotion*	• None provided
Cruz (2013)	The effectiveness of a community health program in improving diabetes knowledge in the Hispanic population: Salud y Bienestar (Health and Wellness)	To examine the impact of a successful 12 month behavioral intervention to improve diabetes control on healthcare utilization in American Samoa	• 1-group pre-test-post-test design	• Hispanic Americans	*Diabetes Empowerment Education Program*	• Not stated	• 2 - 7 days	*Semi- annual refresher trainings*

Sarah (2013)	Impact of a Diabetes Control and Management Intervention on Healthcare Utilization in American Samoa	to present the training process and fidelity of study protocol implementation using a promotora model for community-based diabetes prevention.	• RCT	• American Samoa	• Not stated	• 12 months		• not stated
Sosa (2013)	Lessons Learned from Training of Promotores de Salud for Obesity and Diabetes Prevention	To increase weight loss and glycaemic control using an innovative community health worker (CHW) delivered intervention program in African America women	• Quasi-experimental pre-test, post test study	• Hispanic Americans	• Not stated	• Unclear	*60 hours of training*	• None provided
Cummings (2013)	EMPOWER: a randomized trial using community health workers to deliver a lifestyle intervention program in African American women with Type 2 diabetes: design, rationale, and baseline characteristics.	to explore the impact and feasibility of a pilot Community Health Worker (CHW) intervention to improve diabetes management among Bangladeshi-American individuals with type 2 diabetes living in New York City	• RCT	• African Americans	*Cognitive behavioural technique, small change model of behavioural change*	• 12 months	*50hour community based training on self study, didactic DM mgt*	*ongoing supervision call by investigators assesses knowledge. No other formal follow up training reported*
Islam	Evaluation of a community health worker pilot intervention to improve diabetes management in Bangladeshi immigrants with type 2 diabetes in New York City.	To describe the feasibility of using a community-based participatory research (CBPR) approach to implement the Power to Prevent (P2P) diabetes prevention education curriculum in rural African American (AA) setting	1-group pre-test-post-test design	• Asians *Bangladeshi*	• Not stated	• 12 months	• Not stated	• not stated
Cené	Implementation of the power to prevent diabetes prevention educational curriculum into rural African American communities: a feasibility study.	to examine the impact of a 10-week lifestyle management program delivered by community-based paraprofessionals in improving clinical markers and dietary behaviors related to diabetes control in an underserved population	• 1-group pre-test-post-test design	• African Americans	*Maxwell's 5 M (Model, Mentor, Monitor, Motivate, and Multiply) training mode*	• 12 months	*provided but duration not stated*	• None provided

Saxe-Custack (2013)	A Patient-Centered Approach Using Community- Based Paraprofessionals to Improve Self-Management of Type 2 Diabetes.	To assess whether community health workers (CHWs) could improve glycemic control among Mexican Americans with diabetes	• 1-group pre-test-post-test design	• Hispanics and African Americans *Mixed race including whites*	• Motivational Interviewing *Motivational interviewing techniques as well as adult learning techniques were integral components of the program*	• Less than 6 months	• 2 - 7 days *3 day training of CHW on motivational interviewing and adult learning*	
Rothschild (2014)	Mexican American trial of community health workers: a randomized controlled trial of a community health worker intervention for Mexican Americans with type 2 diabetes mellitus.	To evaluate whether a community health worker (CHW) intervention improved clinically relevant markers of diabetes care in adult Hispanics	• RCT	• Hispanic Americans *Mexican American*	• Cognitive Theory	• 24 months	*100 hours of training on diabetes, behavioral self-management support, and home visit*	• not stated
Palmas (2014)	Results of the northern Manhattan diabetes community outreach project: a randomized trial studying a community health worker intervention to improve diabetes care in Hispanic adults.	To compare a peer leader (PL) versus a community health worker (CHW) telephone outreach intervention in sustaining improvements in HbA1c over 12 months after a 6-month diabetes self-management education (DSME) program.	• RCT	• Hispanic Americans	*Small Steps, Big Rewards framework,*	• 12 months	• Not stated	• not stated
Tang (2014)	Comparative Effectiveness of Peer Leaders and Community Health Workers in Diabetes Self-management Support: Results of a Randomized Controlled Trial	To compare outcomes between community health worker use of a tailored, interactive, Web-based, tablet computer-delivered tool (iDecide) and use of print educational materials	• RCT	• Hispanic Americans	*Behavioural modification strategies. Patient empowerment approach* • Motivational Interviewing	• 18 months	*160 hours of community outreach training plus 80 h of diabetes education, home visit experiences, human subjects tutorial;*	• not stated

Heisler (2014)	Comparison of Community Health Worker–Led Diabetes Medication Decision-Making Support for Low-Income Latino and African American Adults With Diabetes Using E-Health Tools Versus Print Materials	The Neighborhood Health Talker Program used community members trained as cultural health brokers to engage their communities in conversations about "living diabetes well."	• RCT	Hispanics and African Americans	• Motivational Interviewing *Motivational Interviewing-based communication approaches and diabetes self-management support*	• Less than 6 months	*0 hours of training on motivationa lntervieweing*	*4-8 hours of booster training annually*
Cadzow (2014)	"Living well with diabetes": evaluation of a pilot program to promote diabetes prevention and self-management in a medically underserved community.	To determine the effects of a culturally competent diabetes self-management intervention in Mexican Americans with type 2 diabetes.	1-group pre-test-post-test design	• African Americans		• 6 months	• 2 - 7 days *I week formal training on leadership, diabetes knowledge*	• None provided
Carrasquillo (2014)	Rationale and design of the Miami Healthy Heart Initiative: a randomized controlled study of a community health worker intervention among Latino patients with poorly controlled diabetes.		• RCT	• Hispanic Americans *Latinos*	• Community-oriented model	• 12 months	*75 hour training on various aspects of disease mgt.*	*Ongoing periodic CHW training on issues such as CHW skills, clinic and insurance navigation, cardiovascular disease and diabetes care, and human subjects occurred on average about every 2 months*
Pérez-Escamilla (2015)	Impact of a community health workers-led structured program on blood glucose control among Latinos with type 2 diabetes: The DIALBEST Trial	*To examine the impact of the Diabetes Among Latinos Best Practices Trial (DIALBEST), a community health worker (CHW)-led structured intervention for improving glycemic control among Latinos with T2D*	• RCT	• Hispanic Americans	*Problem solving theory and stages of change theory* • Motivational Interviewing	• 18 months	*65 h of core training. More than 25 h of supplemental training*	• not stated

McDermott (2015)	Community health workers improve diabetes care in remote Australian Indigenous communities: results of a pragmatic cluster randomized controlled trial	*to evaluate the effectiveness of a community-based health-worker led case management approach to the care of Indigenous adults with poorly controlled type 2 diabetes*	• RCT	• Aboriginial Australians	• Not stated	• 18 months	• 8 days - 1 month<br>*3 weeks of intensive training on diabetes and other chronic disease management*	*two workshops where they underwent refresher training, including in Good Clinical Practice and reflective practice.*
Wagner (2015)	Community health workers assisting Latinos manage stress and diabetes (CALMS-D): rationale, intervention design, implementation, and process outcomes	*to compare enhanced standard diabetes care with enhanced standard care plus community health worker (CHW) delivered stress management for Latinos with type 2 diabetes*	• RCT	• Hispanic Americans	• Cognitive Theory	• 12 months	• >1 month<br>*"150 hours over 5 months. Including 45hours Diabetes training, Self-management and stress management trainings"*	*Her ongoing training included review- ing and critiquing, with PIWagner, videotapes of her-self delivering DE and SM*
Schmidt (2015)	Impact of a Community Health Worker–Led Diabetes Education Program on Hospital and Emergency Department Utilization and Costs	*To assess the impact of a community health worker–led diabetes education program (DEP) on hospital utilization trends and to evaluate the return on investment*	• Retro-prospective cohort study	• Hispanic Americans<br>*Mexican Americans*	• Not stated	• 12 months	• Not stated	• not stated
