# Supplementary material for: Role of community health workers in type 2 diabetes mellitus self-management: A scoping review
Source: PLoS One. 2018 Jun 1;13(6):e0198424. doi: 10.1371/journal.pone.0198424 (PMC5983553; doi:10.1371/journal.pone.0198424)
Supplement: S2 Appendix — (PDF) [file pone.0198424.s002.pdf]

S2 Appendix

Short Title	Title	Role of CHW	Medium of Intervention	Participants contact duration	Outcome measured	Findings
Allen (2011)	COACH trial: a randomized controlled trial of nurse practitioner/community health worker cardiovascular disease risk reduction in urban community health centers: rationale and design.	• Support<br>• Advocacy			• HbA1c<br>• Blood pressure<br>• Lipids	Study outcome not yet ready at time article was published
Babamoto (2009)	Improving Diabetes Care and Health Measures Among Hispanics Using Community Health Workers: Results From a Randomized Controlled Trial	• Education<br>• Support	• One to one communication<br>• Telephone calls	10 week education sessions	• HbA1c<br>• BMI/Weight<br>• Medication Adherence<br>• Emergency Room visit/Hospitalisation	Improved HbA1c, no change in BMI, reduced ER admission, increased medication adherence
Beckham (2008)	Diabetes management: utilizing community health workers in a Hawaiian/Samoan population	• Education<br>• Support	• One to one communication<br>• Practising practical skill		• HbA1c	Improved HbA1c level
Brown (2002)	Culturally competent diabetes self-management education for Mexican Americans: the Starr County border health initiative.	• Education<br>• Support	• Discussion group session(s)<br>• Practising practical skill	52 contact hours over 12 months. three months of weekly two-hour instructional and (2) six months of biweekly plus three months of monthly two-hour support group sessions	• HbA1c<br>• BMI/Weight<br>• Knowledge about Diabetes<br>• Lipids<br>• Other<br>Fasting Blood sugar	Improved HbA1c, FBS and Diabetes Knowledge. No change in BMI and Lipids

Brown (2012)	Cost-effectiveness analysis of a community health worker intervention for low-income Hispanic adults with diabetes.	• Education<br>• Support<br>• Advocacy	• One to one communication<br>• Practising practical skill<br>• Presentation / lecture	Not stated	• HbA1c<br>• Cost<br>• Other<br>Quality of life Years (QALY)	Better cost effectiveness ratio per QALY gained compared to other studies. Better cost effectiveness for reducing A1c among people with initial higher A1c compared to those with lower A1c
Cadzow (2014)	"Living well with diabetes": evaluation of a pilot program to promote diabetes prevention and self-management in a medically underserved community.	• Education	• Discussion group session(s)<br>• Outreach	The conversations lasted anywhere from 30 minutes to 2 hours and ranged in size from 3 to 15 participants	• Knowledge about Diabetes	
Carrasquillo (2014)	Rationale and design of the Miami Healthy Heart Initiative: a randomized controlled study of a community health worker intervention among Latino patients with poorly controlled diabetes.	• Support	• Discussion group session(s)<br>• One to one communication<br>• Telephone calls	4 initial enactment visits, follow up phone calls and enfd of programme evaluation.	• HbA1c<br>• Medication Adherence<br>• Blood pressure<br>• Lipids<br>• Physical activity	Not documented in this article
Castillo (2010)	Community-based Diabetes Education for Latinos The Diabetes Empowerment Education Program	• Education<br>• Support	• Discussion group session(s)<br>• Presentation / lecture	2-hour group sessions over 10 weeks.	• HbA1c<br>• BMI/Weight<br>• Knowledge about Diabetes<br>• Self-efficacy<br>• Blood pressure	
Cené	Implementation of the power to prevent diabetes prevention educational curriculum into rural African American communities: a feasibility study.	• Education	• Discussion group session(s)	twelve 60–90 minute interactive sessions	• Random blood sugar<br>• BMI/Weight<br>• Blood pressure<br>• Other<br>feasibility of CHW led SM	acceptable to facilitators . Improvements were seen in diabetes knowledge and the impact of healthy eating and physical activity on diabetes prevention, but no significant changes in blood glucose, BP, or weight.

Collinsworth (2013)	Effectiveness of a community health worker-led diabetes self-management education program and implications for CHW involvement in care coordination strategies.	• Education<br>• Support		two initial 60-minute educational sessions and quarterly clinical assessments scheduled for 30 to 60 minutes for a maximum of 6 patient contact hours over 12 consecutive months.	• HbA1c<br>• BMI/Weight<br>• Blood pressure	Statisticaly significang improvement in HbA1c and Systolic blood pressure. No statistically significant differences in body mass index or diastolic blood pressure were observed
Cruz (2013)	The effectiveness of a community health program in improving diabetes knowledge in the Hispanic population: Salud y Bienestar (Health and Wellness)	• Education	• Presentation / lecture<br>one-90 min training session	one-90 min training session	• Knowledge about Diabetes	Improved Diabetes knowledge
Culica (2007)	CoDE: Community Diabetes Education for uninsured Mexican Americans.	• Education<br>• Support<br>• Advocacy		7 patient contact hours over 12 consecutive months.	• HbA1c<br>• BMI/Weight<br>• Blood pressure	Improved HbA1c but no change in BMI or Blood Pressure
Cummings (2013)	EMPOWER: a randomized trial using community health workers to deliver a lifestyle intervention program in African American women with Type 2 diabetes: design, rationale, and baseline characteristics.	• Education<br>• Support	• One to one communication<br>• Telephone calls	16 individual contacts over 12 months	• HbA1c<br>• BMI/Weight<br>• Knowledge about Diabetes<br>• Self-management practice	
Davis (2007)	Teaching How, Not What: The Contributions of Community Health Workers to Diabetes Self-Management	• Education<br>• Support	• One to one communication<br>• Other (specify) email and mail communication<br>• Telephone calls	10-week self-management and 10-week support classes at the clinic as well as patient follow-up via telephone.	• Implementation strategy<br>• Other<br>How do CHW support self-management of diabetes mellitus	The assistance CHWs reported providing was most often in the form of encouragement/motivation.
De Pue (2013)	Nurse–Community HealthWorker Team Improves Diabetes Care in American Samoa	• Education<br>• Support	• Discussion group session(s)<br>• One to one communication	Patients at moderate risk were seen monthly by CHWs and pa- tients at lower risk were seen every 3 months for one year	• HbA1c<br>• BMI/Weight<br>• Self-management practice<br>• Blood pressure	"improvement in HbA1c, No improvement in BMI, BP"

Deitrick (2010)	Understanding the Role of the Promotora in a Latino Diabetes Education Program	• Education<br>• Support				
DePue	Implementation of a culturally tailored diabetes intervention with community health workers in American Samoa.	• Education	• Discussion group session(s)<br>• One to one communication	104 initial visits, 1350 individual follow-up visits, and 61 group sessions (average of 5 participants at each), totaling 1728 contacts	• Other Intervention fidelity, cost of intervention.	Twenty-eight percent of participants moved to a lower risk group over the year. Estimated intervention cost was $656 per person. Participants with less education were more likely to attend optimal percentage of visits
Gary (2003)	Randomized controlled trial of the effects of nurse case manager and community health worker interventions on risk factors for diabetes-related complications in urban African Americans	• Support	• One to one communication<br>• Telephone calls	3 face to face visits lasting 45 minutes or more	• HbA1c<br>• Blood pressure<br>• Lipids	Improvement in HbA1c, Lipids and diastolic blood pressure
Gary (2004)	A randomized controlled trial of the effects of nurse case manager and community health worker team interventions in urban African-Americans with type 2 diabetes	• Support<br>• Advocacy	• One to one communication Home visits<br>• Telephone calls	CHW conducts at least three contacts per participant yearly with at least one of those three being a face-to-face home visit	• HbA1c<br>• Emergency Room visit/Hospitalisation<br>• Blood pressure<br>• Lipids	Not stated
Heisler (2014)	Comparison of Community Health Worker–Led Diabetes Medication Decision-Making Support for Low-Income Latino and African American Adults With Diabetes Using E-Health Tools Versus Print Materials	• Education<br>• Support	• One to one communication<br>• Printed materials / posters<br>• Telephone calls	1 -2 hour session with CHW and 2 follow up phone calls	• HbA1c secondary outcome<br>• Medication Adherence<br>• Knowledge about Diabetes<br>• Self-management practice<br>• Self-efficacy	Improvement in all primary and secondary outcomes.
Henderson (2013)	Community-based participatory research and user-centered design in a diabetes medication information and decision tool.	• Education	• Other (specify)		• Other	

Higgins (2003)	The Promotora Telemedicine Project : Combining Technology and Cultural Sensitivity to Improve Diabetes Care in a Medically Underserved Community	• Support	• Other (specify) Telemedicine		• Cost	No result yet
Hill-Briggs (2007)	Training community health workers as diabetes educators for urban African Americans: value added using participatory methods	• Support			• Other<br>Lessons learned on CHW training	
Ingram (2007)	The impact of promotoras on social support and glycemic control among members of a farmworker community on the US-Mexico border.	• Education<br>• Support<br>• Advocacy	• Discussion group session(s)<br>• One to one communication<br>• Telephone calls	The duration of each contact varied from a 5-minute reminder telephone call to an hour-long educational session or a 2-hour peer support group	• HbA1c<br>• Self-management practice<br>• Blood pressure<br>• Lipids	Improvement in HbA1c, Systolic BP, HDL cholesterol
Islam	Evaluation of a community health worker pilot intervention to improve diabetes management in Bangladeshi immigrants with type 2 diabetes in New York City.	• Education	• Discussion group session(s)<br>• One to one communication	The intervention consisted of 6 monthly, CHW-facilitated 2.5-hour group sessions. Study participants also received 3 one-on-one visits from CHWs at months 3, 6, and 9 of about 60 to 90 minutes in length,	• HbA1c<br>• BMI/Weight<br>• Medication Adherence<br>• Knowledge about Diabetes<br>• Self-efficacy<br>• Lifestyle Change Dietary changes<br>• Physical activity	Improvements were seen in diabetes knowledge, exercise and diet to control diabetes, frequency of checking feet, medication compliance, and self-efficacy of health and physical activity.<br>Reduction in HbA1c
Kegley (2012)	Role of Community Health Workers in Improving Diabetes Outcomes	• Support	• One to one communication	Not stated	• HbA1c<br>• BMI/Weight<br>• Knowledge about Diabetes	improved HbA1c, diabetes knowledge but no change in BMI

Liebman (2007)	Establishing diabetes self-management in a community health center serving low-income Latinos.	• Education<br>• Support	• Discussion group session(s)<br>• One to one communication<br>• Practising practical skill	11 sessions of weekly breakfast club, 11 weekly drop in snack club, 11 sessions of diabetes education classes	• HbA1c<br>• Knowledge about Diabetes<br>• Self-efficacy<br>• Physical activity	Improved HbA1c
Liebman (2008)	Quality Improvement in Diabetes Care Using Community Health Workers	• Support	• One to one communication<br>• Telephone calls		• HbA1c<br>• Other<br>Keeping clinical appointment	Improved glycaemic control. Improved appointment keeping
Lujan (2007)	Promotora diabetes intervention for Mexican Americans.	• Education<br>• Support	• Discussion group session(s)<br>• Telephone calls	8 weekly 2-hour, participative group classes and telephone follow- up to the intervention participants	• HbA1c<br>• Knowledge about Diabetes	Improved diabets knowledge, Improved HbA1c
McDermott (2015)	Community health workers improve diabetes care in remote Australian Indigenous communities: results of a pragmatic cluster randomized controlled trial	• Support	• One to one communication		• HbA1c<br>• BMI/Weight<br>• Blood pressure<br>• Lipids	Improved in HbA1c, better medication use in intervention. group, no in Lipid,difference in BMI or weight between both groups
McElmurry (2009)	Implementation, outcomes, and lessons learned from a collaborative primary health care program to improve diabetes care among urban Latino populations.	• Education	• Discussion group session(s)<br>• One to one communication<br>• Telephone calls		• HbA1c<br>• Self-management practice<br>Blood sugar monitoring	Improvement in HbA1c level, increased blood sugar monitoring.

McEwen	Type 2 diabetes self-management social support intervention at the U.S.-Mexico border.	• Education<br>• Support<br>• Advocacy	• Discussion group session(s)<br>• One to one communication	six monthly 2-hour group sessions and three 60–90 minute individually tailored sessions	• HbA1c<br>• BMI/Weight<br>• Knowledge about Diabetes<br>• Physical activity<br>• Other<br>diabetic distress	The intervention improved behavioral outcomes related to T2DM self-care activities, sedentary behaviors, distress with T2DM regimen, and diabetes knowledge. No imporvement in HbA1c and BMI
Ndou (2013)	A rapid assessment of a community health worker pilot programme to improve the management of hypertension and diabetes in Emfuleni sub-district of Gauteng Province, South Africa.	• Support	• One to one communication		• Random blood sugar<br>• Blood pressure	Blood pressure controlled in participants but blood glucose not controlled in participamts compared to usual care patients
Otero-sabogal (2010)	Physician – community health worker partnering to support diabetes self- management in primary care	• Education<br>• Support	• Discussion group session(s)<br>• One to one communication<br>• Telephone calls		• HbA1c<br>• BMI/Weight<br>• Self-management practice<br>• Blood pressure<br>• Lipids	"improvement in HbA1c, cholesterol, PAM score, No improvement in BMI, BP"
Palmas (2014)	Results of the northern Manhattan diabetes community outreach project: a randomized trial studying a community health worker intervention to improve diabetes care in Hispanic adults.	• Education<br>• Support	• Discussion group session(s)<br>• One to one communication<br>• Telephone calls	4 one- on-one visits, 10 group sessions, and 10 follow-up phone calls per subject, over a 12-month period.	• HbA1c<br>• Blood pressure<br>• Lipids	no improvement in A1C levels in the intervention group. no improvement in the secondary outcomes of blood pressure and LDL-cholesterol levels
Pérez-Escamilla (2015)	Impact of a community health workers-led structured program on blood glucose control among Latinos with type 2 diabetes: The DIALBEST Trial	• Education<br>• Support	• One to one communication<br>• Printed materials / posters	17 home-visit sessions	• HbA1c<br>• Random blood sugar<br>• Blood pressure<br>• Lipids	Improvement in HbA1c and significant effect on fasting glucose There was no significant effect on blood lipid levels, hypertension, and weight

Prezio (2013)	Community Diabetes Education (CoDE) for uninsured Mexican Americans: a randomized controlled trial of a culturally tailored diabetes education and management	• Education<br>• Support<br>• Advocacy	• One to one communication<br>• Printed materials / posters	7 h of contact with the CHW during scheduled appointments over 12 months	• HbA1c<br>• BMI/Weight<br>• Blood pressure<br>• Lipids	"improvement in HbA1c, No improvement in BMI, Blood Pressure"
Rothschild (2014)	Mexican American trial of community health workers: a randomized controlled trial of a community health worker intervention for Mexican Americans with type 2 diabetes mellitus.	• Education<br>• Support	• One to one communication<br>• Other (specify) home visits	36 home visits over 2 years. 90 minutes every forthnight first year then every month next year	• HbA1c<br>• BMI/Weight<br>• Medication Adherence<br>• Self-management practice<br>• Blood pressure<br>• Lipids<br>• Physical activity	Improved HbA1c, no change in medication adherence. Improved physical activity and reduced BMI. Control group had better lipid control
Ryabov (2011)	The impact of community health workers on behavioral outcomes and glycemic control of diabetes patients on the U.S.-Mexico border	• Education<br>• Support	• One to one communication	1 hour monthly visit for 24 months	• HbA1c<br>• BMI/Weight<br>• Knowledge about Diabetes<br>• Self-management practice	The intervention group showed a significant improvement after 2 years of intervention in all outcomes, except Body Mass Index (BMI)
Sarah (2013)	Impact of a Diabetes Control and Management Intervention on Healthcare Utilization in American Samoa	• Education	• One to one communication<br>• Other (specify) flip charts		• HbA1c<br>change in HbA1c by number of intervention visits<br>• Emergency Room visit/Hospitalisation	Increased primary care physician visit. Increased visits lead to reduced HbA1c. No effect on emergency department visit.
Saxe-Custack (2013)	A Patient-Centered Approach Using Community- Based Paraprofessionals to Improve Self-Management of Type 2 Diabetes.	• Education<br>• Support<br>• Advocacy	• Discussion group session(s)<br>• One to one communication	10weeks—one time eachweek for approximately 90minutes	• HbA1c<br>• BMI/Weight<br>• Lifestyle Change<br>• Blood pressure<br>• Physical activity	Improved HbA1c, fruit intake and BMI. No change In physical activity. Differences between baseline and follow-up systolic and diastolic blood pressure were not significant

Schmidt (2015)	Impact of a Community Health Worker–Led Diabetes Education Program on Hospital and Emergency Department Utilization and Costs	• Education<br>• Support	• Presentation / lecture<br>• Other (specify)<br>Periodic assessment	2 initial educational session with up to 6 subsequent clinical assessment sessions of .5 to 1 hour each	• Emergency Room visit/Hospitalisation<br>• Cost<br>• Other<br>Length of stay in hospital	Significant reduction in mean inpatient encounters , reduction in length of stay and reduction in inpatient cost
Sixta (2008)	Texas-Mexico Border Intervention by Promotores for Patients With Type 2 Diabetes	• Education<br>• Support	• Presentation / lecture<br>lectures	The diabetes self-management course was comprised of ten 1.5-hour group sessions held weekly.	• HbA1c<br>• Knowledge about Diabetes	increased knowledge but no changes in health beliefs or HbA1c level over the 6-month period
Sosa (2013)	Lessons Learned from Training of Promotores de Salud for Obesity and Diabetes Prevention	• Education	• Other (specify)<br>Just training		• Other<br>Lessons learned from training CHW for SM support	Post knowledge test scores were high (M=83.8; SD=6.4). Promotores suggested future trainings include more time to develop presentation skills.
Spencer (2011)	Effectiveness of a Community Health Worker Intervention Among African American and Latino Adults With Type 2 Diabetes: A Randomized Controlled Trial	• Education<br>• Support	• Discussion group session(s)<br>• One to one communication	Eleven 2-hour group sessions of 8 to 10 participants were held every 2 weeks and 2 home visits of about 60 minutes each in length per month	• HbA1c<br>• Knowledge about Diabetes<br>• Self-management practice<br>• Blood pressure<br>• Lipids	Improvement in HbA1c and diabetes knowledge in intervention compared to control. No difference in changes in physical activity, LDL cholesterol and BP between the two groups
Sul (2004)	The Involvement of Lay Educators in the Diabetic Control of Type 2 Diabetic Patients	• Education	• One to one communication<br>• Printed materials / posters	at least 3 two-hour sessions over a period of 3 months	• HbA1c<br>• BMI/Weight<br>• Knowledge about Diabetes<br>• Physical activity	Imprvement in HbA1c and physical activity. knowledge, BMI and weight were not significantly different post intervention

Tang (2014)	Comparative Effectiveness of Peer Leaders and Community Health Workers in Diabetes Self-management Support: Results of a Randomized Controlled Trial	• Education<br>• Support	• Curriculum materials Diabetes Self management education<br>• One to one communication Home visits<br>• Telephone calls	two home visits (60 min in length) per month for 6 months	• HbA1c<br>• BMI/Weight<br>• Blood pressure<br>• Lipids	Improveent in HbA1c, waist circumference, but no improvement in BP or lipid levels for CHW supported group
Thompson (2007)	Advancing diabetes self-management in the Mexican American population: a community health worker model in a primary care setting.	• Education<br>• Support<br>• Advocacy	• Curriculum materials<br>• Discussion group session(s)<br>• One to one communication<br>• Outreach<br>• Telephone calls	CHWs contacted participants at least weekly for the first 6 months and monthly thereafter	• HbA1c<br>• BMI/Weight<br>• Lipids	Improved HbA1c, no significant change in BMI and Cholesterol
Treadwell (2010)	Addressing obesity and diabetes among African American men: examination of a community-based model of prevention.	• Education<br>• Support		6 educational sessions (approximately 12 hours) fitness activities (ie, swimming, tennis, boxing, basketball, power walking, etc), totaling approximately 150 hours of collective workout activities completed	• BMI/Weight<br>• Knowledge about Diabetes<br>• Physical activity	
Two (2005)	Racial and Ethnic Approaches to Community Health (REACH) Detroit partnership: improving diabetes-related outcomes among African American and Latino adults.	• Education	• Discussion group session(s)<br>• One to one communication	5 2-hour group meetings delivered every 4 weeks	• HbA1c<br>• Knowledge about Diabetes<br>• Lifestyle Change<br>• Physical activity	Improvement across al measured outcomes

Valen (2012)	An innovative approach to diabetes education for a hispanic population utilizing community health workers	• Education<br>• Support	• Discussion group session(s)<br>• Presentation / lecture<br>• Printed materials / posters		• HbA1c<br>• BMI/Weight<br>• Knowledge about Diabetes<br>• Self-management practice<br>• Self-efficacy	Increased diabetes Knowledge. No increase in Hb1Ac, no change in BMI
Vincent (2007)	A feasibility study of a culturally tailored diabetes intervention for Mexican Americans.	• Education<br>• Support	• Curriculum materials<br>• Discussion group session(s)<br>• Practising practical skill Cooking demonstration, stress management	8 Weekly 2 hour session		Improved knowledge about diabetes disease and improved level of physical activity leading to weight loss among participants
Wagner (2015)	Community health workers assisting Latinos manage stress and diabetes (CALMS-D): rationale, intervention design, implementation, and process outcomes	• Education	• Discussion group session(s)<br>• One to one communication	eight 2-h sessions of group psychoeducation, skills training, and relaxation exercises.	• Knowledge about Diabetes<br>• Self-management practice	Imporovement in Diabetes knowledge.
Walton (2012)	Reducing Diabetes Disparities Through the Implementation of a Community Health Worker–Led Diabetes Self-Management Education Program	• Education<br>• Support<br>• Advocacy	• One to one communication	The CHW scheduled follow-up visits for the patient, in- cluding 2 additional 60-minute visits over an 8-week period to continue the delivery of the DEP curriculum and 4 follow-up visits lasting 30 to 60 minutes at 3, 6, 9, and 12 months for assessment of HbA1c,blood pressure,and BMI.	• HbA1c	Improved HbA1c
